# The effects of extra high dose rate irradiation on glioma stem-like cells

**DOI:** 10.1371/journal.pone.0202533

**Published:** 2018-08-17

**Authors:** Jing Hao, Andrew Godley, Jocelyn D. Shoemake, Zheyi Han, Anthony Magnelli, Jennifer S. Yu

**Affiliations:** 1 Department of Stem Cell Biology and Regenerative Medicine, Cleveland Clinic, Cleveland, Ohio, United States of America; 2 Department of Radiation Oncology, Cleveland Clinic, Cleveland, Ohio, United States of America; 3 Cleveland Clinic Lerner College of Medicine, Cleveland Clinic, Cleveland, Ohio, United States of America; 4 Burkhardt Brain Tumor and Neuro-Oncology Center, Cleveland Clinic, Cleveland, Ohio, United States of America; Northern University, UNITED STATES

## Abstract

Radiation therapy is an integral part of treatment for patients with glioblastoma. New technological advances in linear accelerators have made extra-high dose rate irradiation possible. This shortens patient treatment time significantly compared to standard dose rate irradiation, but the biologic effects of extra high dose rate irradiation are poorly understood. Glioma stem-like cells (GSCs) are resistant to standard radiation and contribute to tumor progression. Here, we assess the therapeutic effect of extra high dose rate vs. standard dose rate irradiation on GSCs. GSCs were exposed to 2, 4 and 6 Gy X-irradiation at dose rates of 4.2 Gy/min or 21.2 Gy/min (400 monitoring units (MU)/min or 2100 MU/min). We analyzed cell survival with cell growth assays, tumorsphere formation assays and colony formation assays. Cell kill and self-renewal were dependent on the total dose of radiation delivered. However, there was no difference in survival of GSCs or DNA damage repair in GSCs irradiated at different dose rates. GSCs exhibited significant G1 and G2/M phase arrest and increased apoptosis with higher doses of radiation but there was no difference between the two dose rates at each given dose. In a GSC-derived preclinical model of glioblastoma, radiation extended animal survival, but there was no difference in survival in mice receiving different dose rates of radiation. We conclude that GSCs respond to larger fractions of radiation, but extra high dose rate irradiation has no significant biologic advantage in comparison with standard dose rate irradiation.

## Introduction

Glioblastoma multiforme (GBM) is the most malignant primary brain tumor with few long term survivors [1]. Standard treatment includes surgical removal of the tumor followed with radiotherapy and chemotherapy [2–3]. Recent technological advances in linear accelerators have permitted treatment of patients with extra high dose rates. The use of extra high dose rate irradiation has shortened treatment time, improving quality of life for patients who are often symptomatic from their cancer. It also improves patient throughput, which is critical in underdeveloped areas where the number of patients needing radiation far exceeds the number of radiation facilities. However, whether extra high dose rate irradiation may confer a radiobiological benefit is unclear.

There have been several reports comparing the biological effects of high dose rate and standard dose rate irradiation. These studies either used low dose rate γ-irradiation generated from radioactive isotopes or X-rays generated from linear accelerators. One study reported that low dose rate irradiation reduced cell survival, caused significant G1 and G2/M cell cycle arrest and increased apoptosis in A549 and H1299 non-small cell lung cancer cell lines [4]. Others found that dose rate did not have a biologically significant effect on cell survival or DNA damage repair in glioblastoma cell lines U87-MG and T98G; cervical cancer cell line SiHa; lung carcinoma cell line H460 and hamster lung cell line V79 [5–6]. In contrast, Sarojini et al. reported that extra high dose rate irradiation at 2400 monitoring units (MU)/min for total dose of 0.5 Gy significantly killed more melanoma cells than 400 MU/min dose rate to the same total dose by inducing more apoptosis and greater DNA damage [7]. Whether these biologic differences exist at clinically significant doses is poorly understood.

Radiation therapy is currently the most effective nonsurgical treatment in glioblastoma management. Unfortunately, tumor recurrence is unavoidable and patients typically recur within 6–9 months of treatment [8]. Glioblastoma contain a heterogeneous mix of cells. Some cells are endowed with an increased ability to resist conventional radiation and chemotherapy and possess a high capacity for self-renewal. These cells, termed glioma stem-like cells (GSCs) or tumor initiating cells, are capable of initiating tumors in vivo and recapitulating the phenotype of the original tumor [9–12].

GSCs play an important role in tumor progression after radiation therapy because they can selectively activate DNA damage checkpoint pathways and enhance DNA damage repair [13–14]. Even though focal irradiation can reduce tumor bulk, surviving GSCs can expand and reinitiate the tumor, and eventually lead to clinically significant tumor recurrence. Finding effective means to target GSCs will improve the durability of tumor control. The current application of high dose rate irradiation in clinic may provide a therapeutic advantage over standard dose rate irradiation by improving the efficiency of GSC kill. Here, we interrogate the effects of high dose rate irradiation on GSC eradication and DNA damage repair using a clinical linear accelerator.

## Materials and methods

### Cell lines and cell culture

GSCs were isolated from xenografts and functionally characterized as previously described [15–16]. Briefly, 2×10^4^ GSCs were implanted into the right frontal lobes of 4- week old female BALB/c nude mice. Mice were euthanized and necropsied when they showed signs of neurologic decline or poor performance status. Animals were anesthetized and underwent cardiac perfusion with PBS. Tumors were disaggregated using the Papain Dissociation System (Worthington Biochemical) according to the manufacturer’s instructions. Isolated cells were recovered in stem cell medium (Neurobasal—a medium with B27 supplement, 40 ng/ml EGF and 40 ng/ml FGF) for at least 6 hrs and then sorted by magnetic cell sorting for GSCs using the surface marker CD133 (Miltenyi Biotec). GSCs were cultured in stem cell medium as described above. GSCs were tested for expression of stem cell markers (Oligo2, Sox2 and Nestin) and lack of expression of the differentiation marker (GFAP). Limiting dilution assays were performed both in vitro and in vivo to confirm the self-renewal and tumor initiation ability of the GSCs [15–16]. All animals were housed in the AAALAC-accredited animal facility with temperature, humidity and lighting controlled. Animal care was monitored daily by certified veterinary staff and/or approved laboratory personnel. Every effort was made to minimize discomfort, distress, pain or injury to the mice. All mice were maintained in accordance with the applicable portions of the Animal Welfare Act and the guidelines set by the IACUC. Animal experiments were approved by the Institutional Animal Care and Use Committee at Cleveland Clinic, protocol 2015–1482.

### Cell viability assays

1×10^3^ irradiated GSCs were plated into each well of 96-well plates. Cell titers were determined after the indicated number of days using the Cell Titer-Glo Luminescent Cell Viability Assay kit (Promega). Day 0 data was collected right after the irradiation. Luminescent readings in each group were repeated six times and normalized to day 0 and presented as mean ± SD.

### Tumorsphere formation assay

1×10^3^ irradiated GSCs were plated into each well of 96-well plates. On day 7 tumorspheres were counted in four repeats and representative pictures were taken with EVOS XL core cell imaging system. Data were presented as mean ± SD.

### Colony assay

2×10^4^ irradiated GSCs were plated into each well of 6-well plates. On day 7, cells were fixed and dyed with crystal violet solution (0.05% crystal violet, 1% formalin and 10% methanol in distilled water) overnight at room temperature. Cells were washed and the plates were dried in ambient air. Colonies were counted and presented as mean ± SD.

### Immunofluorescence

Immunofluorescence of cells was performed as described [17]. Briefly, GSCs were seeded on Matrigel coated cover slips for 24 hrs before subjected to X-irradiation. Cells were first pre-extracted with 0.2% Triton in PBS on ice for 5 min followed by 4% paraformaldehyde fixation for 10 mins at room temperature (RT). Cells were blocked with 10% BSA for 30 mins and incubated in 1:3000 γ-H2AX antibody (Millipore, Cat#05–636) at 4°C overnight followed by the appropriate secondary fluorescently labeled antibodies (Invitrogen Molecular Probes) for one hour at RT. Nuclei were counterstained with DAPI. Images were taken with a wide-field fluorescence microscope (Leica). Three independent experiments were performed. The data were presented as mean ± SD.

### Cell cycle analysis

Cells were collected 24 hrs after irradiation. Cells were digested with Accutase solution (Thermo Fisher, Cat# A1110501) and fixed in 70% ethanol. The fixed cells were stored at -20°C for at least 24 hrs. On the day of analysis cells were centrifuged and washed with PBS. Cell pellets were dispensed in Propidium Iodide Solution (Thermo Fisher, Cat# F10797) and incubated at room temperature for 1 hr before the flow cytometry analysis. Three independent cell cycle data of each GSCs were analyzed with Modfit LT software and presented as mean ± SD. Cell cycle phases in irradiated cells were compared with controls for statistical significance.

### Apoptosis analysis

Cells were collected 24 hrs after irradiation. Cell lysates were prepared in RIPA buffer (150 mM NaCl, 1.0%NP40, 0.5% sodium deoxycholate, 0.1% SDS, 50 mM Tris, pH 8.0.). Cleaved-Parp (Cell Signaling, Cat# 5625) and cleaved-Caspase 3 (Cell Signaling, Cat# 9661) were used as apoptosis markers.

### Orthotopic mouse xenografts

Intracranial transplantation of 387 GSCs to establish GBM xenografts was performed as described [17–19]. GSCs expressed luciferase to facilitate non-invasive imaging. Briefly, 2X10^4^ cells were implanted into the right frontal lobes of Nod Scid Gamma mice (Jackson Laboratories). Tumors were monitored by IVIS 100 bioluminescent imaging. When the mouse tumors exhibited similar levels of bioluminescence, mice were randomized to control or radiation to 10 Gy in 5 fractions at dose rates of 400 MU/min or 1500 MU/min. Animals were immobilized with ketamine:xylazine cocktail for the radiation. Animals received whole head irradiation on a clinical linear accelerator (Novalis TX) using half-beam block technique, and a 1 cm water-equivalent bolus was used to improve dosimetry. Additional lead shielding was used to reduce body irradiation. The setup was confirmed by light field. Animals were maintained until manifestation of neurological signs or for 180 days post-transplantation.

### Delivery of X- irradiation on a linear accelerator

**Fig 1:**
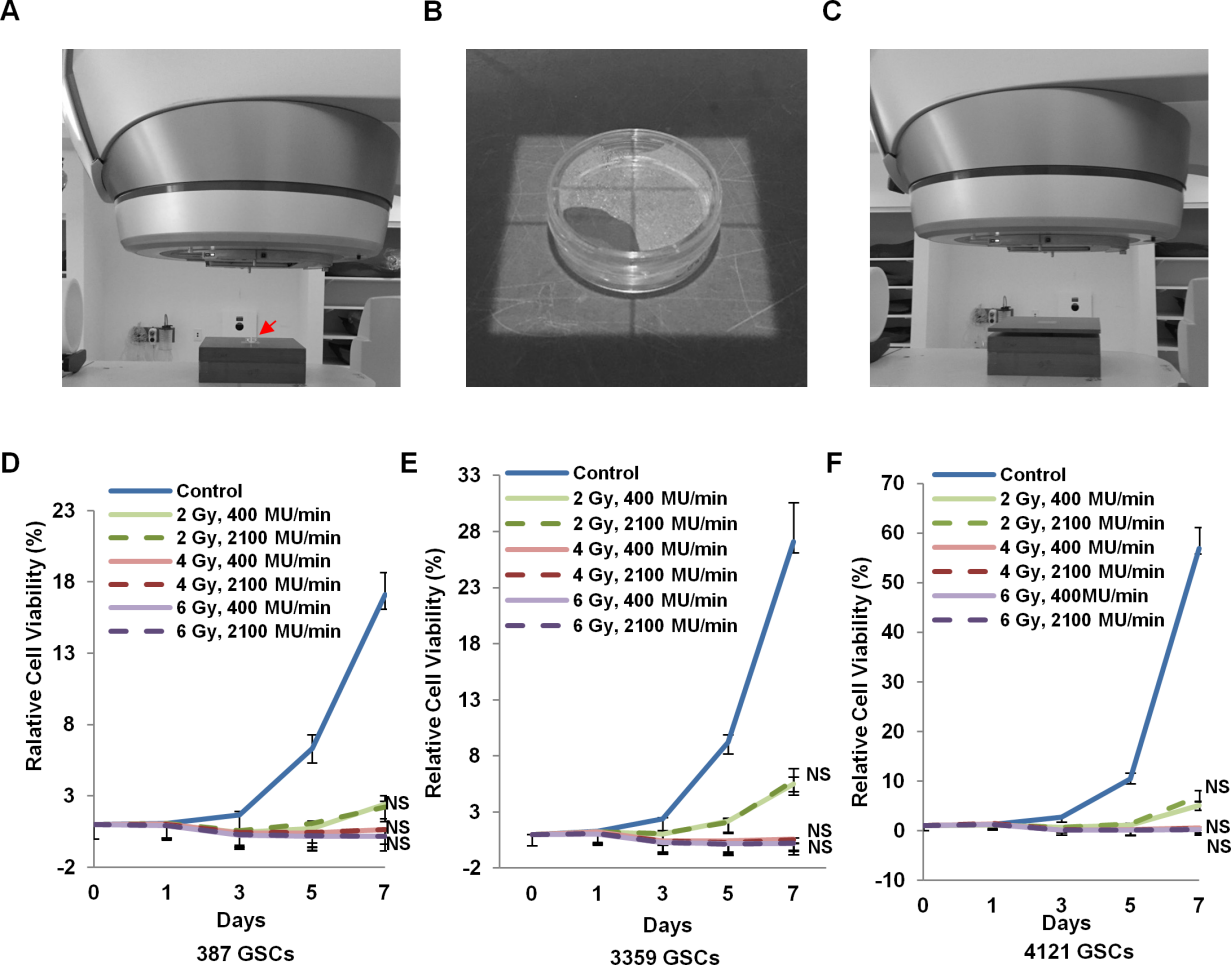
Irradiation set-up and cell viability assay. A. The cell culture dish (red arrow) was placed on solid water to improve dosimetry. B. The cell culture dish was placed at the field center and its position confirmed with crosshairs and the light field. C. Solid water bolus of 1 cm thickness was placed over the cell culture dish to improve dosimetry. (D) 387 GSCs, (E) 3359 GSCs and (F) 4121 GSCs were irradiated to doses of 2, 4 and 6 Gy X- irradiation at dose rates of 400 MU/min or 2100 MU/min. Data presented as mean ± SD. NS, not significant.

Radiation was delivered on clinical linear accelerators (Varian Edge or Novalis Tx) (Fig 1A). A solid water block was placed on the irradiation table under the cells (shown by a red arrow) to provide backscatter. The cell culture plate was placed at the field center given by the light field (Fig 1B). There was 1 cm culture medium in the cell culture plate, and a 1 cm water-equivalent bolus was used to deliver the appropriate dose to the cells (Fig 1C). Cells were treated with a 7x7 cm open field with 6 MV photons to the prescribed dose (2 Gy, 4 Gy, 6 Gy in a single fraction) at a dose rate of 400 MU/min or 2100 MU/min (high dose rate using a flattening filter free mode of the 6 MV beam). A source to surface distance (SSD) of 100 cm was used for the 400, 200 and 100 MU/min dose rates. For the effective 20 MU/min dose rate, an SSD of 224 cm was used with the linear accelerator set at 100 MU/min. An SSD of 80 cm was used to increase the effective dose rate of 2100 MU/min (Varian Edge) or 1500 MU/min (Novalis Tx). The linear accelerator dose rates of 400 MU/min translates to 4.2 Gy/min and 2100 MU/min translates to 21.2 Gy/min in the cells.

### Statistical analysis

All grouped data are presented as mean ± SD. Mantel-cox test were used to analyze the animal survival data. Unpaired t-test was used for all other statistical analyses.

## Results

### Cell viability, tumorsphere formation and colony formation

In order to explore whether extra high dose rate irradiation has therapeutic advantages in killing GSCs, we used three GSC populations in cell viability assays after treatment with doses of 2, 4 and 6 Gy. The viability of all GSC populations was dramatically hindered in a dose-dependent manner (Fig 1D–1F). There was no difference in cell viability when GSCs were irradiated with 400 MU/min or 2100 MU/min at the three different doses. These results suggest that extra high dose rate irradiation does not confer significant benefits over standard dose rate irradiation in terms of GSC eradication. Similarly, analysis of lower dose rates of 400, 200, 100 and 20 MU/min also did not affect cell viability (S1 Fig).

**Fig 2:**
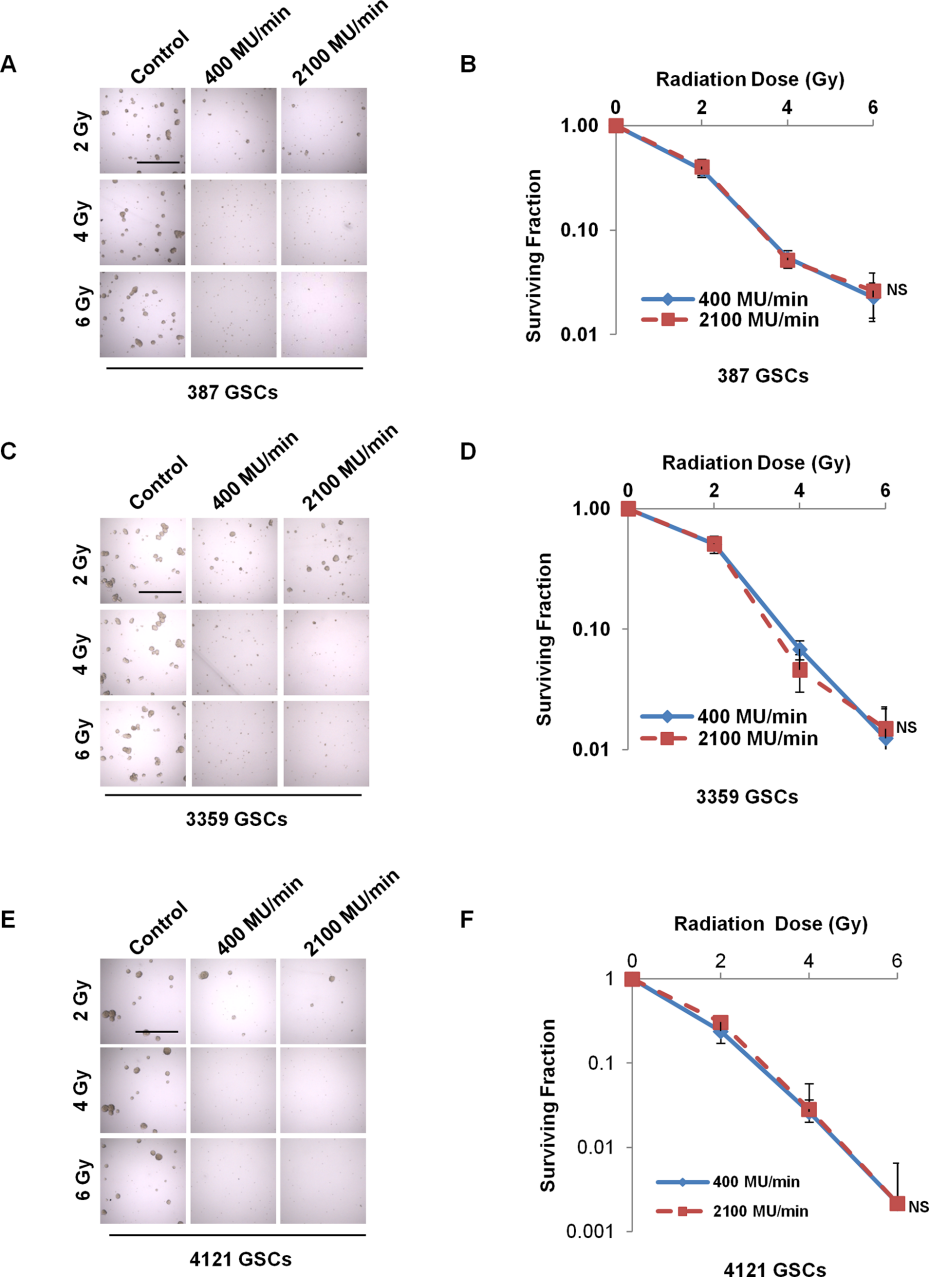
Tumorsphere formation of GSCs treated with different dose rates of radiation. Representative bright field images of 387 GSCs (A), 3359 GSCs (C) and 4121 GSCs (E) irradiated at the indicated dose and dose rates (400 MU/min or 2100 MU/min). Quantification of the surviving fraction of 387 GSCs (B), 3359 GSCs (D) and 4121 GSCs (F) is shown. There was no statistically significant effect on tumorsphere formation between dose rates at each radiation dose level. Scale bar measures 1000 μm. NS, not significant.

Tumorsphere formation is an indicator of self-renewal of GSCs. We examined the ability of GSCs to form tumorspheres 7 days after irradiation. In general, there were fewer and smaller tumorspheres after irradiation in all three GSC populations (Fig 2A, 2C and 2E). Cell survival curves of GSCs treated at dose rates of 400 MU/min and 2100 MU/min overlapped (Fig 2B, 2D and 2F), suggesting similar effects on tumorsphere formation with standard and extra high dose rate irradiation.

**Fig 3:**
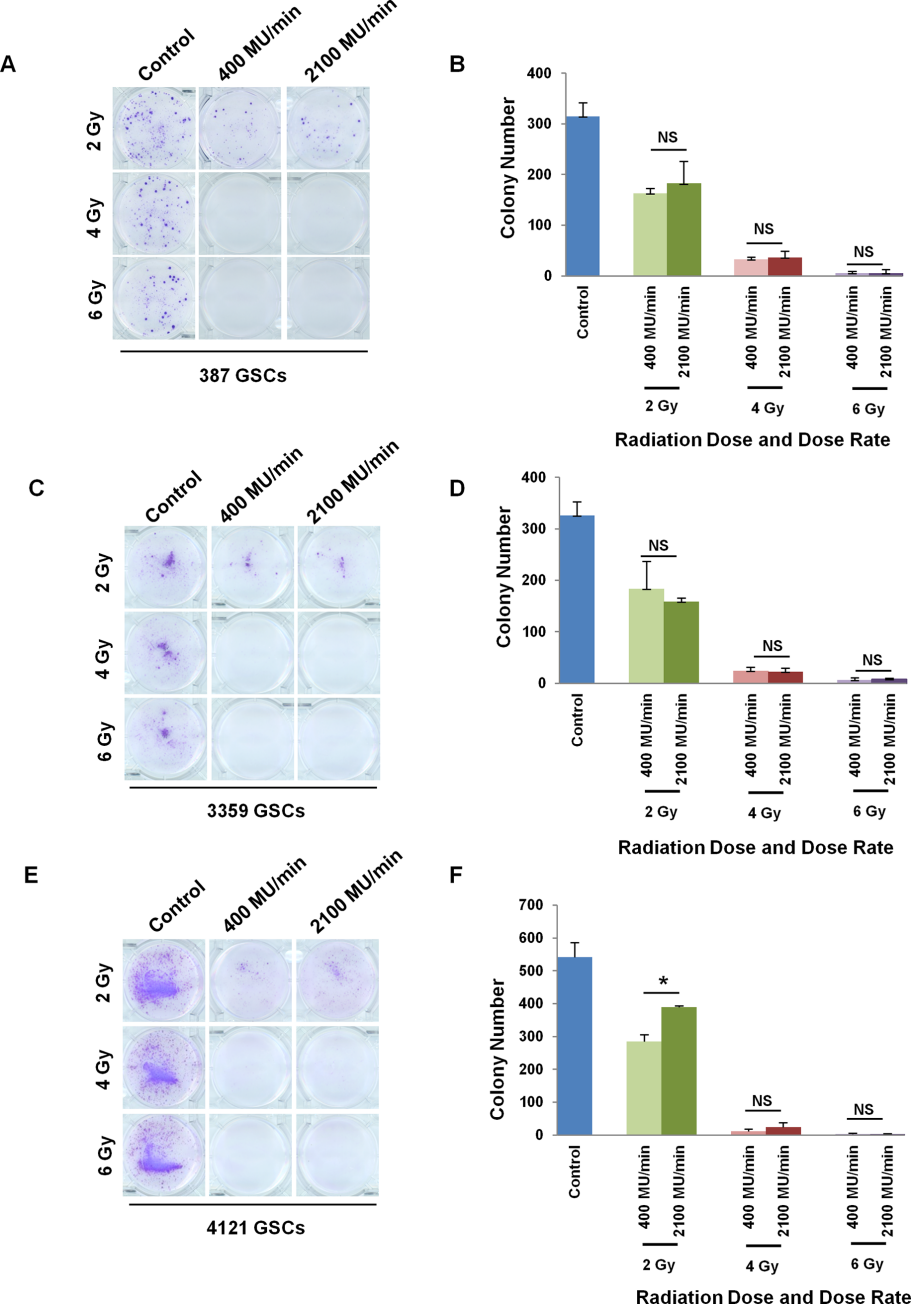
Colony formation assay of GSCs treated with different dose rates of irradiation. Bright field images of 387 GSCs (A), 3359 GSCs (C) and 4121 GSCs (E). Quantification of colony numbers in 387 GSCs (B), 3359 GSCs (D) and 4121 GSCs (F) is shown. Data presented as mean ± SD. *, p<0.05; NS, not significant.

A complementary study of colony formation was also assessed. GSCs were plated in Matrigel-coated dishes to facilitate adherence to the plate, then irradiated, fixed and stained with crystal violet on day 7 post-irradiation. As seen in Fig 3, there were no significant differences in colony formation at all doses examined. We observed a slightly higher colony number in 4121 GSCs irradiated with 2 Gy (p< 0.05) with 2100 MU/min (Fig 3E and 3F) but not in the other two GSC populations (Fig 3A–3D). Overall, there was no evidence that these dose rates differentially impacted colony formation. Consistent with cell viability assays, GSCs displayed dose-dependent responses to the total dose of radiation.

### γ-H2AX foci formation

**Fig 4:**
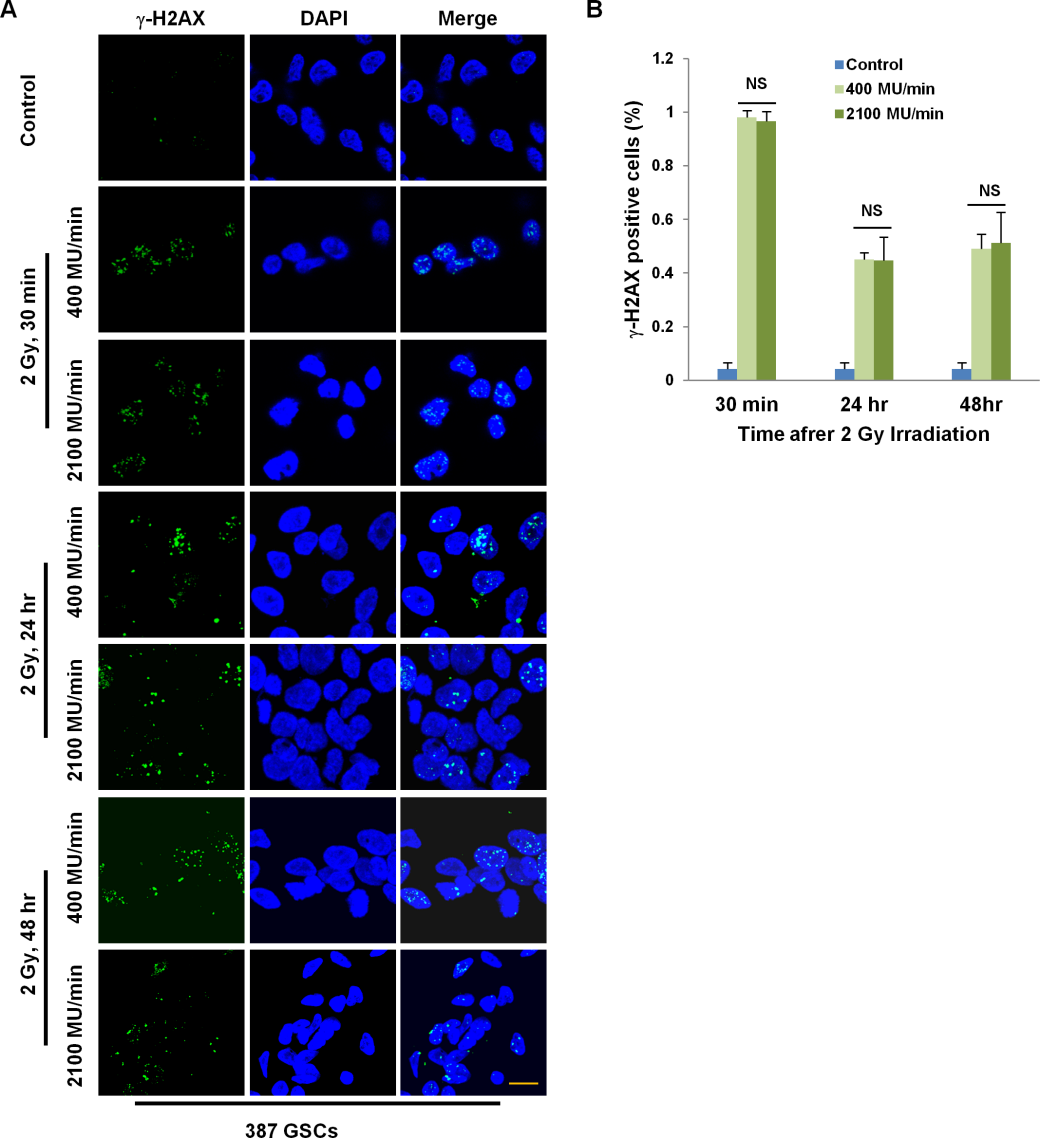
γ-H2AX foci formation in GSCs. (A) Representative immunofluorescence images of 387 GSCs harvested at 30 min, 24 hrs and 48 hours after irradiation with the indicated dose and dose rates. (B) Quantification of γ-H2AX foci from three independent experiments presented as mean ± SD. There was no statistically significant difference in γ-H2AX foci between dose rates at each radiation dose level. Scale bar 20 μm. NS, not significant.

Formation of DNA double strand breaks is a major mechanism in radiation-induced cell death. We monitored DNA double strand break resolution by γ-H2AX foci staining in GSCs irradiated to 2 Gy at dose rates of 400 MU/min or 2100 MU/min at serial time points. As expected, γ-H2AX foci staining was strong in all cells 30 min after irradiation and the number of foci per cell was similar regardless of the dose rate delivered (Fig 4A). At 24 and 48 hrs after irradiation, approximately 50% GSCs showed distinct γ-H2AX foci. There was no difference in γ-H2AX foci formation between the two dose rates with each dose (Fig 4B). These data suggest that standard and extra high dose rate irradiation induces similar levels of DNA double strand breaks and that the efficiency of repair is similar.

### Cell cycle distribution

**Fig 5:**
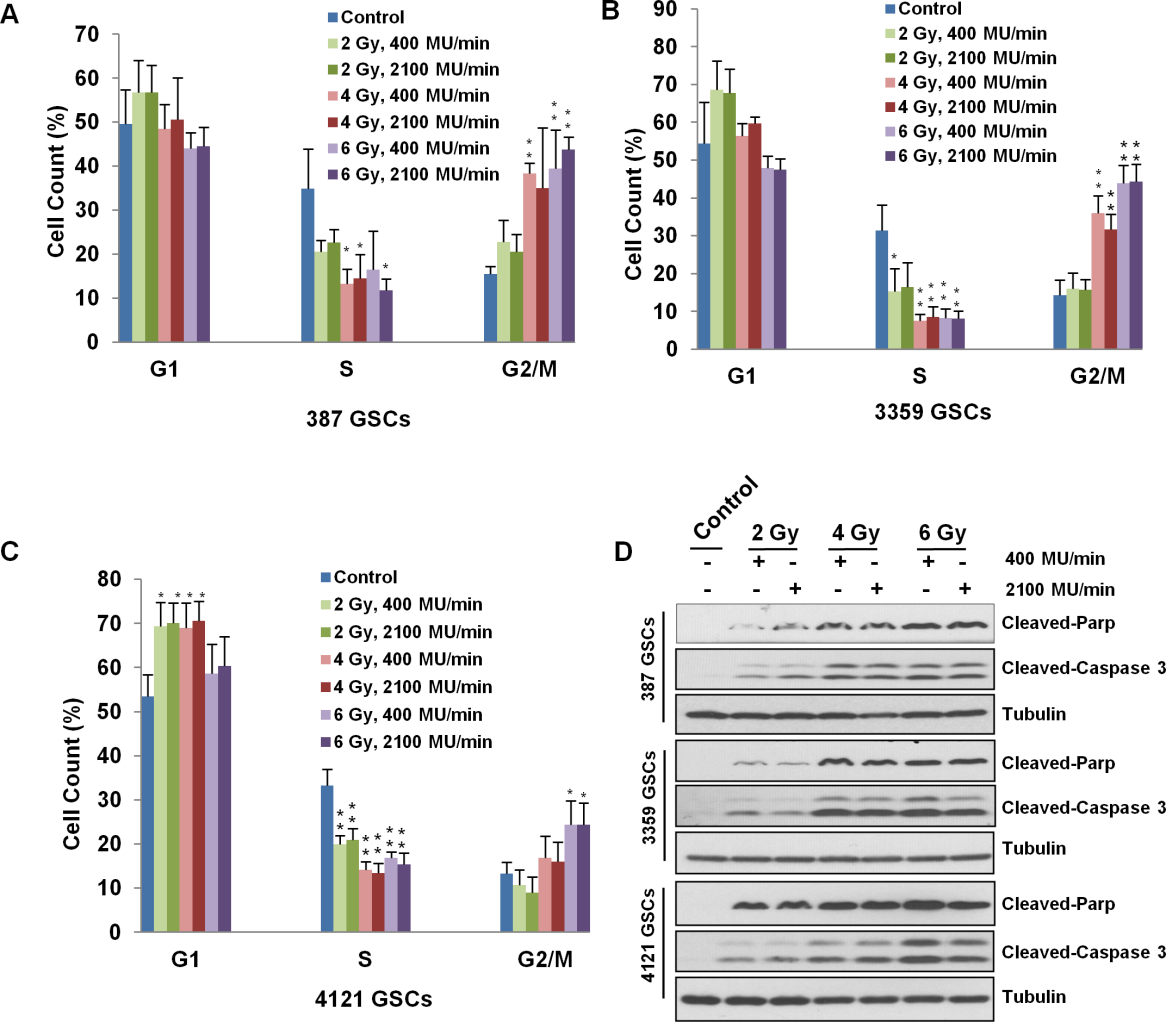
Cell cycle analysis and apoptosis marker expression in irradiated GSCs. (A) 387 GSCs, (B) 3359 GSCs and (C) 4121 GSCs were irradiated to doses of 2, 4 and 6 Gy X-ray at dose rates of 400 MU/min or 2100 MU/min. Cells were collected and fixed with ethanol 24 hrs after irradiation. Three independent experiments were performed for statistical analyses. Data presented as mean ± SD. Statistical significance was calculated in comparison with non-irradiated controls (*, p<0.05; **, p<0.01). There was no statistically significant effect in cell cycle phase between dose rates at each radiation dose level. Apoptosis markers cleaved-Parp and cleaved-Caspase 3 were detected in irradiated GSCs (D). 387, 3359 and 4121 GSCs were subjected to doses of 2, 4 and 6 Gy X-irradiation at dose rates of 400 MU/min or 2100 MU/min. Cells were collected and cell lysates were prepared 24 hrs after irradiation.

Cell cycle analysis was performed 24 hrs after irradiation. In 387 and 3359 GSC populations, G2/M phase arrest was more prominent (Fig 5A and 5B). In 4121 GSCs, 2 and 4 Gy irradiation induced G1 phase arrest while 6 Gy irradiation induced G2/M phase arrest (Fig 5C). The proportion of cells in S phase was significantly smaller in all three GSC populations (Fig 5A–5C). Even though we observed radiation induced cell cycle block in all three GSCs, consistent with prior reports [20], we did not find any significant difference in cell cycle arrest between the two dose rates.

### Apoptosis analysis

We used two markers of apoptosis, cleaved-Parp and cleaved-Caspase 3, to evaluate apoptotic response after irradiation in the three GSC populations. We observed a dose-dependent increase in both cleaved-Parp and cleaved-Caspase 3 (Fig 5D) in GSCs, consistent with the literature [21]. There were no apparent differences in the expression levels of cleaved-Parp or cleaved-Caspase 3 between the two dose rates.

## Animal survival assay

**Fig 6:**
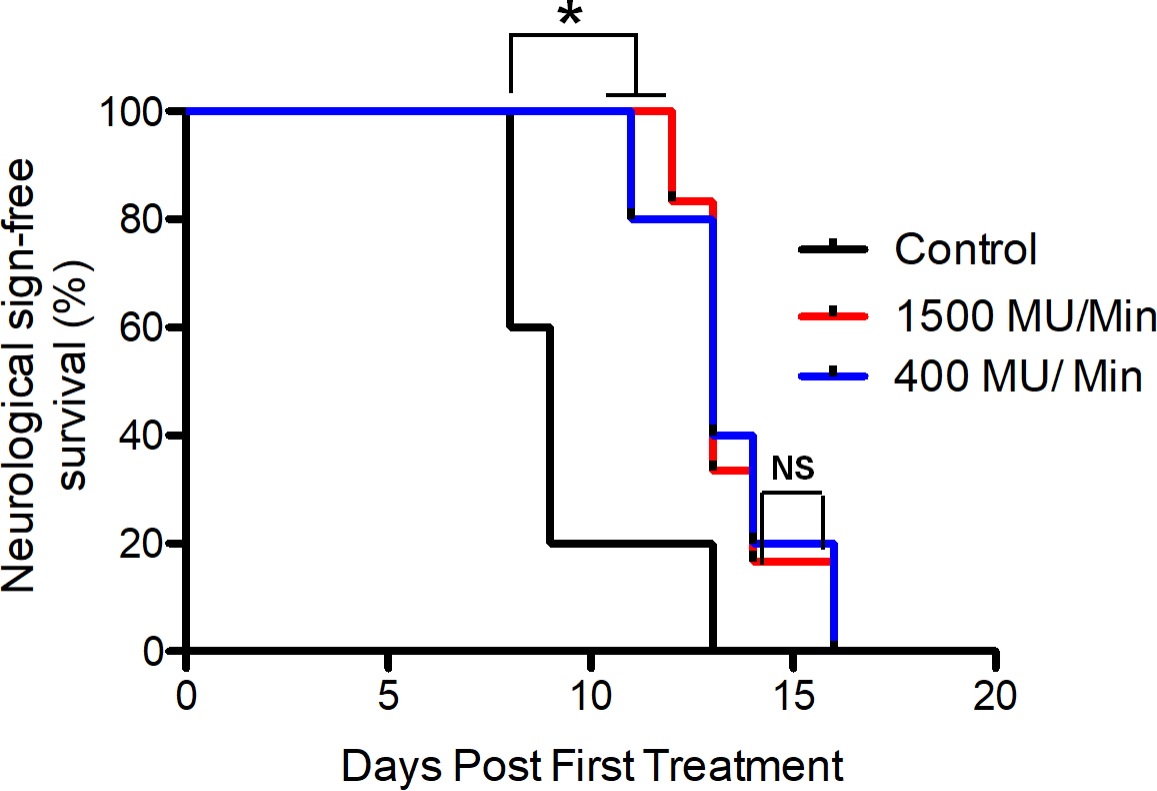
Radiation therapy increases the overall survival in an orthotopic GSC-derived model of glioblastoma. Kaplan-Meier survival curve of animals treated with different dose rates of radiation or control. Mantel-cox test was used to analyze the data. Control N = 5; 1500 MU/min N = 6; 400 MU/min N = 5. Control V.S. irradiated: *, p<0.05; 1500 MU/min vs. 400 MU/min: NS, not significant.

To assess the in vivo effects of extra high dose rate irradiation, we established intracranial xenografts with GSCs. Mice were randomized to control or irradiation at standard or high dose rates. Due to technical limitations, the highest dose rate that we could achieve was 1500 MU/min. Radiotherapy significantly improved the overall survival of mice compared to control non-irradiated mice (Fig 6). However, there was no difference in survival in animals that received radiation at the 2 different dose rates.

## Discussion

Current reports on the biological effects of extra high dose rate irradiation are contradictory and have only been examined using bulk cancer cell lines. Some reports demonstrate that extra high dose rate irradiation is therapeutically advantageous in melanoma cell lines and T98 or U87 glioblastoma cell lines [7, 22], while other reports show that there is no difference in cell death observed in the same glioblastoma cell lines [5, 23].

Our results show the impact of dose rates on the most radioresistant tumor subpopulation, the GSCs [13]. GSCs sit at the apex in the cellular hierarchy of GBM cells. Multiple cell surface markers are used to enrich for GSCs, and multiple markers have been proposed to isolate GSCs [24]. However, identification of GSCs remains functional. GSCs are multipotent and have the ability to differentiate into multiple lineages. Using in vivo limiting dilution assays, even small numbers of GSCs can establish tumors efficiently, with the resulting tumor displaying complex features of the original tumor [24]. The GSCs used in this study have been validated functionally [16]. Importantly, compared to non-GSCs, GSCs are highly resistant to radiation [13, 25–26]. Whereas, non-GSCs can be killed off efficiently with conventional radiation, GSCs persist and therefore play an important role in cancer recurrence [13].

Our study suggests that in GSCs, extra high dose rate irradiation in clinically achievable dose rates has no biologic advantage compared to standard dose rate irradiation. Using complementary assays to assess cell survival, self-renewal, DNA damage repair, cell cycle block and apoptosis, GSCs respond similarly regardless of the dose rates used. Additionally, in a preclinical GSC-derived mouse model of glioblastoma, we failed to detect a survival benefit with high dose rate irradiation. Our data support that GSCs respond to radiation in a dose-dependent manner. Our study suggests that the use of large fractions of radiation over standard fractionation may confer better GSC control. Increasing the dose rate does not appear to improve the response of GSCs to radiation. Nevertheless, high dose rate irradiation improves the overall patient experience and efficiency of treatment delivery and therefore plays an important role in facilitating patient care.

## Supporting information

S1 Fig GSC survival assay in low dose rate range. 3691 GSCs were irradiated with 2 Gy X-ray at 400, 200, 100 and 20 MU/min dose rates. Data presented as mean ± SD. There is no statistical difference in cell survival between dose rates. NS, not significant.
(PDF)
