# Supplementary material for: The effects of extra high dose rate irradiation on glioma stem-like cells
Source: PLoS One. 2018 Aug 17;13(8):e0202533. doi: 10.1371/journal.pone.0202533 (PMC6097670; doi:10.1371/journal.pone.0202533)
Supplement: S1 Fig — 3691 GSCs were irradiated with 2 Gy X-ray at 400, 200, 100 and 20 MU/min dose rates. Data presented as mean ± SD. There is no statistical difference in cell survival between dose rates. NS, not significant. (PDF) [file pone.0202533.s001.pdf]

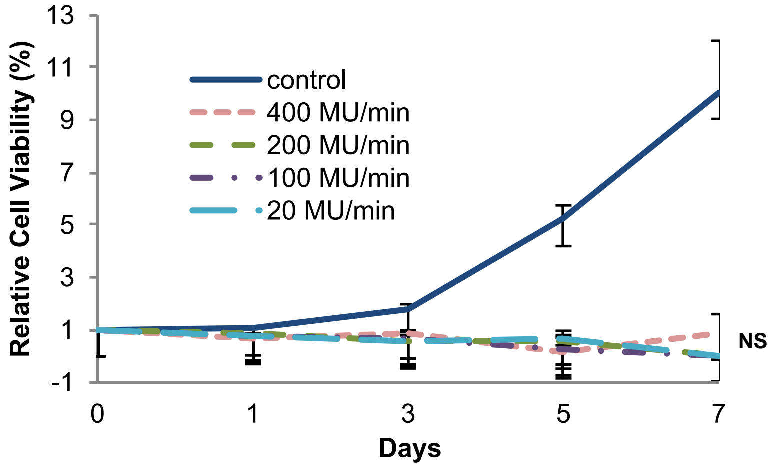

3691 GSCs irradiated with 2 Gy

S1 Fig. GSC survival assay in low dose rate range. 3691 GSCs were irradiated with 2 Gy X-ray at 400, 200, 100 and 20 MU/min dose rates. Data presented as mean ± SD. There is no statistical difference in cell survival between dose rates. NS, not significant.
